# Promoter and domain structures regulate FLA12 function during Arabidopsis secondary wall development

**DOI:** 10.3389/fpls.2023.1275983

**Published:** 2023-11-16

**Authors:** Yingxuan Ma, Julian Ratcliffe, Antony Bacic, Kim L. Johnson

**Affiliations:** ^1^ La Trobe Institute for Agriculture & Food, Department of Animal, Plant and Soil Science, AgriBio Building, La Trobe University, Bundoora, VIC, Australia; ^2^ State Key Laboratory of Tree Genetics and Breeding, Co-Innovation Center for Sustainable Forestry in Southern China, Nanjing Forestry University, Nanjing, China; ^3^ Key Laboratory of Forest Genetics and Biotechnology of Ministry of Education, Nanjing Forestry University, Nanjing, China; ^4^ Sino-Australia Plant Cell Wall Research Centre, College of Forestry and Biotechnology, Zhejiang Agriculture and Forestry University, Hangzhou, China

**Keywords:** Arabidopsis thaliana, fasciclin-like arabinogalactan proteins (FLAs), glycosylphosphatidylinositol-anchor (GPI-anchor), interfascicular fibre (IF), xylem vessel (XV), N-glycosylation, O-glycosylation, secondary cell wall (SCW)

## Abstract

**Introduction:**

Fasciclin-like arabinogalactan-proteins (FLAs) are a family of multi-domain glycoproteins present at the cell surface and walls of plants. *Arabidopsis thaliana* FLA12 and homologs in cotton, *Populus*, and flax have been shown to play important functions regulating secondary cell wall (SCW) development. FLA12 has been shown to have distinct roles from the closely related FLA11 that also functions during SCW development. The promoter and domain features of FLA12 that regulate functional specificity have not been well characterized.

**Methods:**

In this study, promoter swap experiments of *FLA11* and *FLA12* were investigated. Mutation of proposed functional regions within FLA12 were used to investigate the role of post-translational modifications on sub-cellular location and trafficking. Domain swap experiments between FLA11 and FLA12 were performed to identify regions of functional specificity.

**Results:**

Promote swap experiments showed that *FLA12* is differentially expressed in both stem and rosette leaves compared to FLA11. Post-translational modifications, in particular addition of the glycosylphosphatidylinositol-anchor (GPI-anchor), were shown to be important for FLA12 location at the plasma membrane (PM)/cell wall interface. Domain swap experiments between FLA11 and FLA12 showed that the C-terminal arabinogalactan (AG) glycan motif acts as a key regulatory region differentiating FLA12 functions from FLA11.

**Discussion:**

Understanding of FLA12 promoter and functional domains has provided new insights into the regulation of SCW development and functional specificity of FLAs for plant growth and development.

## 1 Introduction

Arabinogalactan-proteins (AGPs) are glycoproteins that have been identified in cell walls of plant and algal species and encoded by large and diverse multigene families. A feature that defines AGPs are arabinogalactan (AG) glycomotif sequences that direct addition of type II AG glycan chains *O*-linked to hydroxyproline (Hyp) in the protein backbone. The surrounding amino acids alanine (Ala), serine (Ser) and threonine (Thr) were hypothesized to be necessary for proline (Pro) to be hydroxylated to Hyp (Showalter et al., 2010). AG glycans have been shown to be added to Hyp when it occurs in a non-contiguous manner such as “Ser-Hyp-Thr-Hyp” (Tan et al., 2003). The basic structure of AG glycans are β-1,3-galactan backbones with β-1,6-galactan sidechains terminated by arabinose, 4-O-methyl-glucopyranosyl, fucose and xylose (Tryfona et al., 2012). Studies suggest terminal 4-O-methyl-glucopyranosyl residues are important for AGPs to regulate calcium signaling at the cell surface (Lopez-Hernandez et al., 2020). AGPs are involved in many aspects of plant growth and development including reproduction and seed development, root hair growth, stem biomechanics and tolerance to abiotic stresses (Ellis et al., 2010; Leszczuk et al., 2023; Xue et al., 2017; Ma et al., 2018; Zhou et al., 2022; Lopes et al., 2023). The fasciclin-like AGPs (FLAs), are a subclass of AGPs that contain a fasciclin1 (FAS1) domain in addition to regions containing AG glycomotifs (Johnson et al., 2003; Shafee et al., 2020). The FAS1 domain has been identified in a wide range of taxa including bacteria, insects, mammals and plants and shown to have cell adhesion related functions (Bastiani et al., 1987; Elkins et al., 1990; Kim et al., 2000; Clout et al., 2003; Johnson et al., 2003; Takayama et al., 2006).

The number of FLAs in angiosperms varies with most having 20-40 members expressed in a range of tissues (Guerriero et al., 2017; Shafee et al., 2020). FLAs are predicted to be located at the plasma membrane, attached by glycosylphosphatidylinositol (GPI)-anchors, or in the wall. Distinct members appear to have tissue specificity, for example *FLA11* and *FLA12* in *Arabidopsis thaliana* and homologs in eucalyptus, poplar, cotton, salix, jute, hemp and *Nicotiana benthamiana* are highly expressed during secondary cell wall (SCW) development and predicted to regulate SCW composition in response to mechanical stresses (MacMillan et al., 2010; MacMillan et al., 2015; Guerriero et al., 2017; MacMillan et al., 2017; Wang et al., 2017; Hossain et al., 2020).

SCWs are deposited between the plasma membrane (PM) and primary cell walls (PCWs) following the cessation of cell expansion in specific cell types such as xylem vessels (XVs) and interfascicular fibres (IFs). SCWs play several roles, including transport of water and nutrients and mechanical support. Glycoproteins, including FLAs, are minor components of SCWs yet have been shown to influence biomechanical properties. Arabidopsis *fla11 fla12* double mutant stems had lower cellulose content with higher microfibril angle (MFA) and reduced stem tensile strength and tensile modulus elasticity (MacMillan et al., 2010). *FLA6-antisense* plants in poplar showed defects in tension wood formation (Lafarguette et al., 2004; Wang et al., 2017). SCW composition and structures can change during growth and in response to stress. Previous studies showed that the closely related *FLA11* and *FLA12* likely respond to different mechanical stress signals to regulate SCWs (Ma et al., 2022). Differences in expression pattern between *FLA11* and *FLA12* have been observed; in young stems, *proFLA12*::YFP-FLA12 signals are preferentially located in the IF region and *proFLA11*::YFP-FLA11 signals are mostly associated with xylem tissue (MacMillan et al., 2010; Ma et al., 2022). In previous studies we showed that overexpression of *FLA11* (OE-FLA11) can trigger initiation and development of SCWs but not OE-FLA12, suggesting functional specificity (Ma et al., 2022). The domain features that regulate FLA11 have recently been characterized (Ma et al., 2023), whereas the promoter and domain features that distinguish FLA12 remain unclear.

Post-translational glycosylation including *N*-glycosylation, *O*-glycosylation, and GPI-anchor attachment are important for the structural integrity, location, and biological functions of many plant glycoproteins, including FLAs (Strasser et al., 2021). FLA11 and FLA12 proteins are predicted to contain a N-terminal signal peptide, two AG-glycan motifs flanking a single FAS1 domain (containing *N*-glycosylation motifs), and a GPI-anchor at the C-terminus (Johnson et al., 2003; Shafee et al., 2020; Ma et al., 2022). Both AG glycans and GPI-anchors were suggested to regulate FLA trafficking to the PM and location at PM or in the wall (Xue et al., 2017). The AG glycans could reinforce transport of FLAs to the outer leaflet of the plasma membrane (PM) due to protein-carbohydrate and/or carbohydrate-carbohydrate interactions within PM microdomains (Yeats et al., 2018). In previous studies, the C-terminal AG glycan motif of FLA11 was identified as important for its function during SCW development (Ma et al., 2023). The GPI-anchor has proven important for AGP synthesis and secretion (Bernat-Silvestre et al., 2021). Removal of the GPI-anchor signal sequence of FLA4 inhibited secretion (Xue et al., 2017), and lack of a GPI-anchor signal sequence affected location and function of FLA11 (Ma et al., 2023). GPI-anchors can associate with membrane domains rich in sphingolipids and sterols, termed lipid rafts or membrane microdomains proposed to traffic in distinct vesicles for delivery to the apical surface (van de Meene et al., 2017). The importance of GPI-anchor attachment for the function of FLA12 in SCWs is yet to be determined. The role of these glycosylation modifications in regulating FLA12 secretion/location and biological functions also remains unclear.

In this study, we performed promoter swap experiments and showed that the different expression patterns between *FLA12* and *FLA11* play a role in their functional specificity. Using domain mutation/deletion variants, we found the GPI-anchor was important for FLA12 secretion and location at the PM/cell wall interface. Domain swap experiments were further used to characterize FLA12 domain features and confirmed that the C-terminal AG is important for differentiating FLA12 functions from FLA11. These results provide new insights into the regulation of SCW development and the putative cell surface sensing pathways.

## 2 Materials and methods

### 2.1 Plant material

Arabidopsis WT (Col-0), OE-FLA12, and OE-FLA12 mutant/domain swap plants were grown under long day (16 h light/8 h dark) conditions at 22/18 °C in controlled environment rooms. *N. benthamiana* plants were grown in standard glasshouse conditions under long day (16 h light/8 h dark) conditions at 25/20 °C. The light intensity for Arabidopsis and tobacco growth was 120 µM/cm^2^/s.

### 2.2 Generation of vectors and transgenic plants

A pGreenII0179 vector backbone (Hellens et al., 2000) was used for constructing *proFLA12*::His-YFP-FLA12 and *proFLA12*::His-YFP-FLA12 mutant variant vectors as used in previous study (Ma et al., 2022). The yellow fluorescence protein (YFP) used in this study is VENUS (Rekas et al., 2002). **Supplementary Tables S1** and **S2** provide the list of vectors and primers used for FLA12 domain mutation/deletion. **Supplementary Tables S3**, **S4** provide the list of vectors and primers used for FLA12 domain swaps. NEBuilder HiFi DNA Assembly kit (NEW ENGLAND Biolabs, NOTTING HILL Victoria, Australia) was used to construct vectors according to the manufacturer’s instructions. All vectors were confirmed by sequencing and then transformed into *Agrobacterium* strain AGL1. *Arabidopsis* plants were transformed using the flower dip method (Clough and Bent, 1998). Plants were then screened with hygromycin (35 mg/L).

The number of insertions and transgene copies (TC) were predicted based on segregation ratios in the T2 and T3 generation as outlined in Ma et al., 2023. Lines with 70%-85% survival ratio (3:1) in T2 were used for checking YFP signals in primary root vascular tissues. Plants from at least three independent T2 lines were transferred into soil and taken to the T3 generation for further phenotypic analysis. Morphological analysis of plants, including stem, silique and leaf length and width were performed on three biological replicates from three independent transformed lines. Statistically significant differences were determined using Student’s *t*-test, *p* < 0.05.

### 2.3 Transient expression of proteins in *N. benthamiana* leaf

*N. benthamiana* leaf was infiltrated with *Agrobacterium* (OD_600 = _1.0 of each transformed vectors and p19) for imaging (Zeng et al., 2016). YFP (excitation 514 nm, emission 535 nm, detection 517-553 nm), α-ManI-ECFP (excitation 405 nm, emission 480 nm, detection 463-497 nm), and PIP2A-mCherry (excitation 594 nm, emission 632 nm, detection 604-660 nm) signals were detected in infiltrated *N. benthamiana* leaf of 2-3 days post infiltration using Zeiss LSM 780 laser scanning confocal microscope (Oberkochen, Germany).

### 2.4 Histological analysis

Fresh stems were hand-sectioned and stained with Toluidine blue, phloroglucinol-HCl or Mäule reagent to visualize cell walls with an Olympus BX53 microscope (Tokyo, Japan) under bright field (Mitra and Loqué, 2014). At least three plant stems from three independent transformed lines were sectioned and measured for tissue organization analyses at the same developmental stages. Data are shown as mean ± SD. Student’s *t*-test was used for significance analysis with *p* < 0.05.

### 2.5 Transmission-electron microscopy and immunolabelling

A 2 mm region at the base of stems at growth 6.9 (Boyes et al., 2001) when flowering complete, were chemically fixed, dehydrated, and embedded in LR white (Wilson and Bacic, 2012). Thin sections (~80 nm) were acquired for antibody labelling and post-stained (Wilson and Bacic, 2012). For antibody labelling, samples were incubated with anti-6x-His tag monoclonal antibodies (Invitrogen, # MA1-21315, Carlsbad CA, USA) with 1:100 dilutions for 2 h at room temperature. Samples were then washed and incubated with goat anti-mouse 18 nm gold conjugated secondary antibody (Jackson Immuno Research #115-215-166, West Grove, PA, USA) with 1:20 dilutions for 1 h at room temperature. Detection of ultrastructure, HIS-YFP-FLA12 and HIS-YFP-FLA12mutGPI subcellular location was performed for three biological replicates. Grids were imaged using a Jeol 2100 EM microscope (Tokyo, Japan) equipped with a Gatan Orius SC 200 CCD camera (Pleasanton, CA, USA). Gold signals were quantified manually from TEM images and data shown as mean ± SD.

### 2.6 Cell wall analysis

Alcohol insoluble residue (AIR) was prepared from Arabidopsis stems (Pettolino et al., 2012). The Updegraff method was used for crystalline cellulose content measurement (Updegraff, 1969). Acetyl bromide method was used to detect lignin content according to (Chang et al., 2008). Three biological replicates from three independent transformed lines were measured. Data represent mean ± SD. Student’s *t*-test was used for significance analysis with *p* < 0.05.

### 2.7 Protein blotting

Total proteins were extracted from 10-day-old Arabidopsis seedlings using extraction buffer (100 mM Tris-HCl, pH 8.8, 150 mM NaCl, 1mM EDTA, 10% (v/v) glycerol and 1X cOmplete protease inhibitor cocktail (Sigma, #11697498001, St. Louis, MO, USA). GFP-trap (ChromoTek, #gtma20, Bayern, Germany) was used to enrich proteins according to the manufacturer’s instructions. Denatured proteins were used for SDS-PAGE and protein blotting analysis with anti-GFP (Invitrogen, #MA5-15256, Carlsbad CA, USA). Two biological replicates from two independent transformed lines were performed.

## 3 Results

### 3.1 *FLA12* expression predominates in rosette leaves compared to *FLA11*

**Figure 1:**
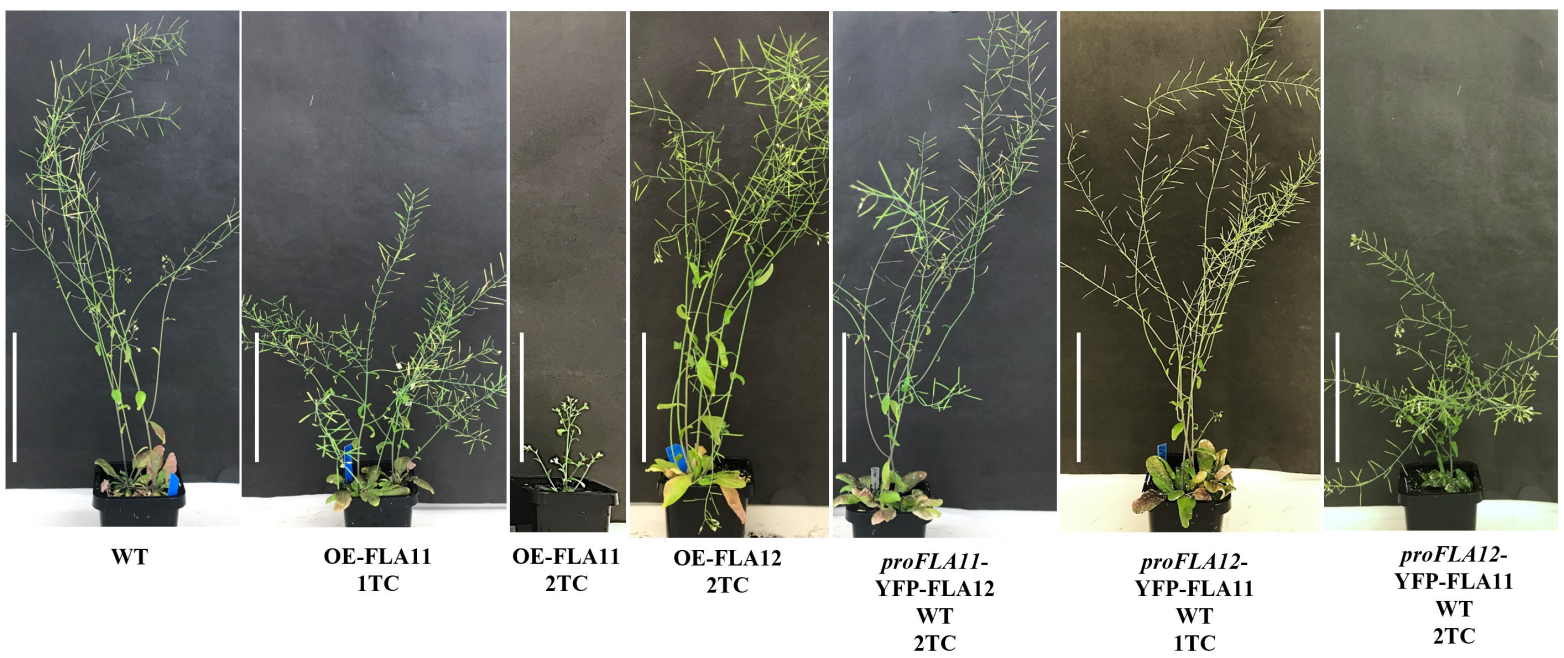
Phenotypes of wild type (WT), OE-FLA11, OE-FLA12, *proFLA11*::YFP-FLA12 WT and *proFLA12*::YFP-FLA11 WT plants. OE-FLA11 plants show reduced stem length in a dosage dependent manner whereas OE-FLA12 show similar phenotype to WT. Promoter swap of *FLA11* and *FLA12* showed that *proFLA12*::YFP-FLA11 WT plants with two transgene copies (2TC) shorter stem length compared to WT (see **Table 1** for quantifications). Scale bar = 10 cm.

In previous studies, the expression patterns of *FLA11* and *FLA12* were shown to differ in young stems (Ma et al., 2022). *FLA11* was shown to have higher expression levels in xylem vessels (XVs) than *FLA12*, whereas *FLA12* expression levels were higher in interfascicular fibres (IFs) (Ma et al., 2022). To investigate how the different *FLA* expression patterns relate to SCW development and plant growth, we conducted promoter swap experiments between *FLA11* and *FLA12*. Expression of *proFLA11*::YFP-FLA12 and *proFLA12*::YFP-FLA11 proteins was confirmed by YFP signals present in XVs of roots (**Supplementary Figure S1**). Phenotyping of *proFLA11*::YFP-FLA12 WT plants showed no obvious plant growth phenotypes compared to wild-type (WT) plants (**Figure 1**) or secondary wall development phenotypes related to OE-FLA11 as shown in our previous study (Ma et al., 2023). Phenotyping analysis of *proFLA12*::YFP-FLA11 WT plants with one or two transgene copies (TC) showed that *proFLA12*::YFP-FLA11 had moderate effects on plant stem and silique length compared with OE-FLA11 (*proFLA11*::YFP-FLA11) that has significantly reduced stem length (**Figure 1**; **Table 1**) (Ma et al., 2022).

**Table 1:** Morphological analyses of wild type (WT), OE-FLA11, OE-FLA12, and *proFLA12:*:YFP-FLA11 WT plants with one or two transgene copies (TC).

Phenotypes	WT	OE-FLA11 1TC	OE-FLA11 2TC	OE-FLA12 2TC	*proFLA12*::YFP-FLA11 WT 1TC	*proFLA12*::YFP-FLA11 WT 2TC
Stem length (cm)	45.4 ± 2.2	24.5 ± 1.2*	7.0 ± 1.0*	40.4 ± 2.2*	43.1 ± 2.1	23.6 ± 5.8*
Silique length (cm)	1.5 ± 0.1	1.1 ± 0.3*	0.6 ± 0.1*	1.7 ± 0.2	1.3 ± 0.1*	1.1 ± 0.1*
Leaf length (cm)	3.6 ± 0.4	2.6 ± 0.4*	1.5 ± 0.2*	3.5 ± 0.4	3.0 ± 0.4*	2.2 ± 0.4*
Leaf width (cm)	2.0 ± 0.3	1.4 ± 0.1*	0.7 ± 0.1*	1.8 ± 0.2	1.9 ± 0.1	1.5 ± 0.2*
Leaf length/width	1.8 ± 0.2	1.9 ± 0.2	2.0 ± 0.1	2.0 ± 0.1	1.6 ± 0.2*	1.5 ± 0.3*

Data shown as mean ± SD. N = 3 plants from three independent transformed lines. Asterisks indicates statistically significant difference, p < 0.05 using Student’s t-test.

**Figure 2:**
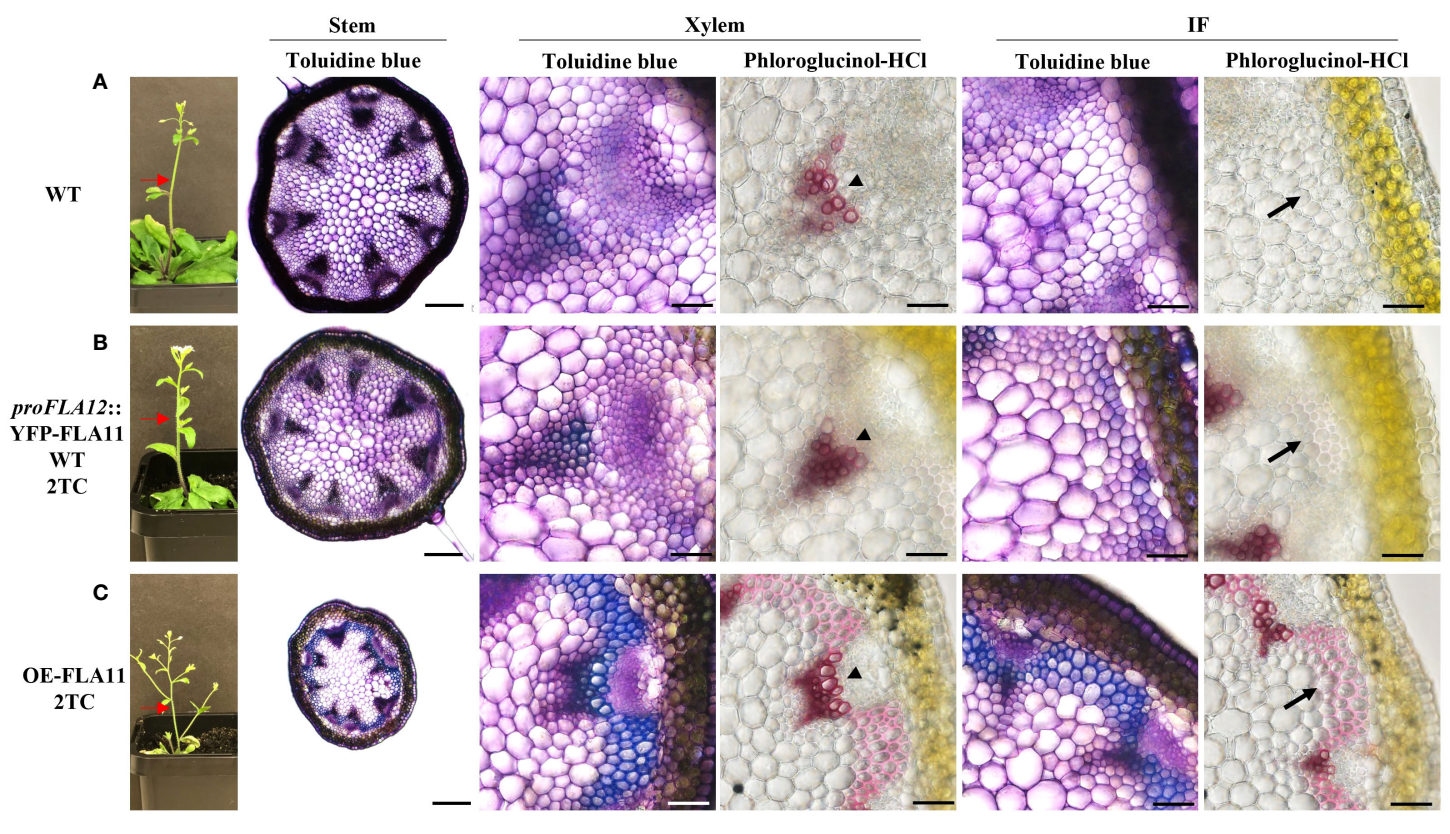
Plant morphology and histology of young stems of WT, *proFLA12*::YFP-FLA11 WT, and OE-FLA11 plants. Fresh stems of WT **(A)**, *proFLA12*::YFP-FLA11 WT with two transgene copies (TC) **(B)**, and OE-FLA11 2TC **(C)** plants at growth stage 6.0 (Boyes et al., 2001) were sectioned at 2 cm below stem top (indicated by red arrows) and stained with Toluidine blue O or phloroglucinol-HCl. OE-FLA11 stems showed early development of metaxylem SCWs compared to WT (indicated by arrow heads). Both *proFLA12*::YFP-FLA11 WT and OE-FLA11 stems showed early development of SCWs in interfascicular fibre **(IF)** cells (indicated by black arrows) compared to WT. Scale bar = 200 µm for stem and 20 µm for xylem and IF.

Consistent with previous studies (Ma et al., 2022), histology analysis of young stems showed that OE-FLA11 can trigger early SCW development in both XV and IF cells. Phenotypic analysis of young stems (stage 6.0; Boyes et al., 2001) showed that *proFLA12*::YFP-FLA11 plants exhibited earlier development of SCW in IFs compared to WT and later development than OE-FLA11 (**Figure 2**). Histological analysis of stage 6.9 mature stems was performed to investigate the development of xylem and morphology of mature XVs. *proFLA12*::YFP-FLA11 2TC plant stems had a similar number of XVs in each xylem bundle compared to WT (**Supplementary Figure S2**). In comparison, the diameters of XVs in *proFLA12*::YFP-FLA11 2TC plant stems were significantly smaller compared to OE-FLA11 and WT plants (**Supplementary Figure S2**).

**Figure 3:**
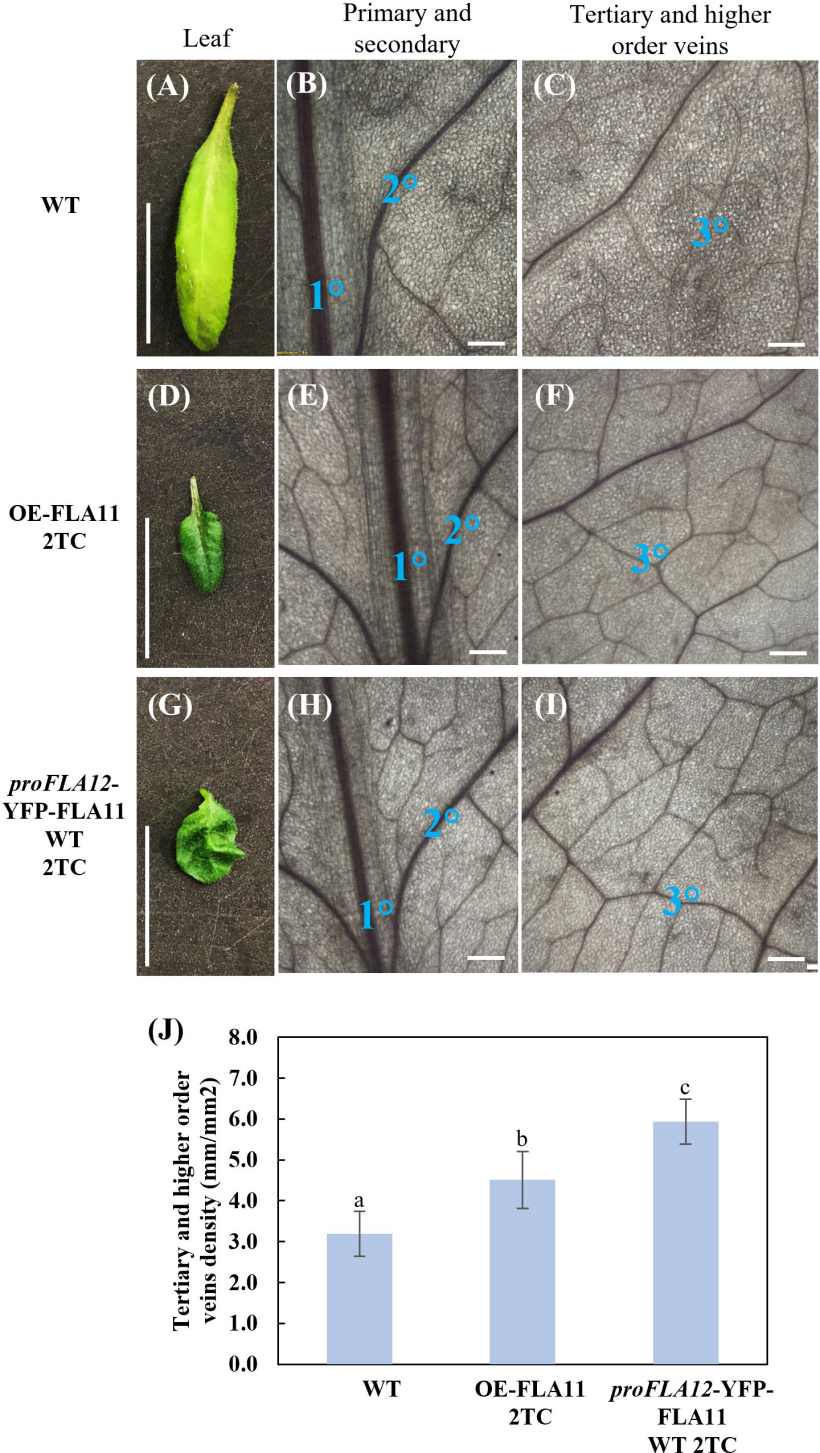
Rosette leaf phenotypes of wild type (WT), OE-FLA11, and *proFLA12*::YFP-FLA11 WT plants. **(A–I)** *proFLA12*::YFP-FLA11 WT plants with two transgene copies (2TC) showed reduced leaf length and length:width ratio with ruffled leaf phenotype (see **Table 1**). **(J)** The density of tertiary and higher order veins in *proFLA12*::YFP-FLA11 WT 2TC plants is higher than WT and OE-FLA11 2TC plants. a, b, c indicates all three groups are significantly different from one another using One-way ANOVA analysis, *p* < 0.05. Scale bar = 1 cm.

In addition to stem phenotypes, *proFLA12*::YFP-FLA11 WT 2TC plants showed reduced rosette leaf length, a smaller length/width ratio, and ruffled leaf phenotype that were not observed in OE-FLA11 or WT plants (**Figure 3**; **Table 1**). Analysis of leaf vein patterns showed that in rosette leaves of *proFLA12*::YFP-FLA11 2TC the density of tertiary and higher order veins was increased compared to OE-FLA11 and WT (**Figure 3**). These results suggest that coordination of differential expression patterns of *FLA11* and *FLA12* is important for the timing and development of SCWs in stems and in rosette leaves.

### 3.2 C-terminal AG motif is the major region that differentiate FLA12 functions from FLA11

**Figure 4:**
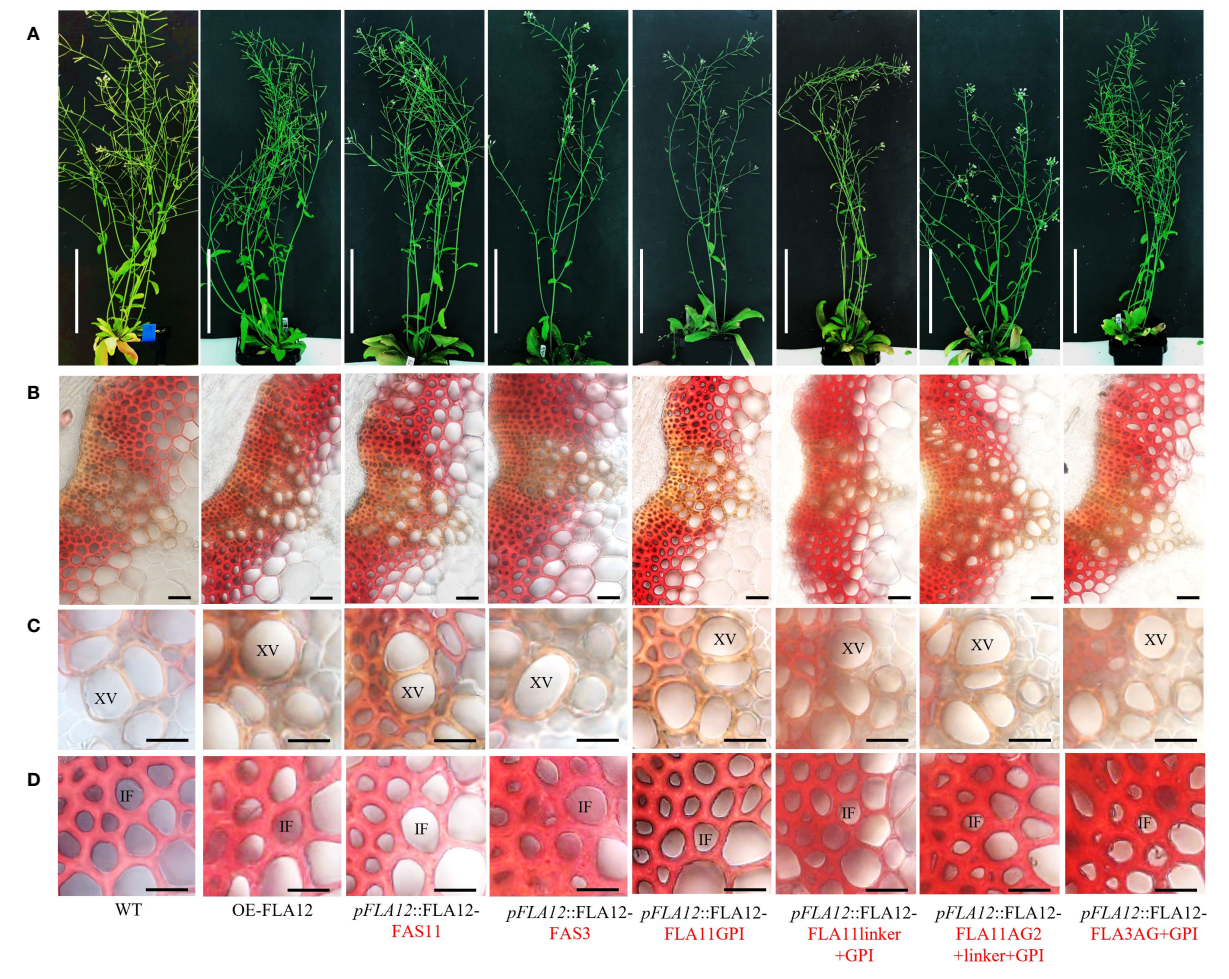
Comparison of FAS1, AG2, linker, and GPI-anchor domain swap plants. **(A)** Representative images of stage 6.9 (Boyes et al., 2001) plant morphology of OE-FLA12, *pFLA12*::FLA12-FAS11, *pFLA12*::FLA12-FAS3, *pFLA12*::FLA12-FLA11GPI, *pFLA12*::FLA12-FLA11linker+GPI, *pFLA12*::FLA12-FLA11AG2+linker+GPI, and *pFLA12*::FLA12-FLA3AG+GPI. (see **Table 2** for stem length quantification, see **Supplementary Figure S3** for schematic representation of domain swaps). **(B–D)** Histological analyses of the base of stems **(B)**, xylem vessels (XVs) **(C)**, and interfascicular fibres (IFs) **(D)** cell morphology and wall lignin composition. Sections taken from fresh stems of stage 6.9 plants at 1 cm from the base were stained with Mäule reagent. Scale bar = 10 cm in **(A)**, 20 µm in **(B)**, 10 µm in **(C, D)**. Red text indicates domains swapped.

To identify if domain features influence FLA12 function, OE-FLA12 protein domains were swapped with corresponding regions from FLA11 or FLA3 (**Supplementary Figure S3**). The fasciclin (FAS) domain of FLA3 (type F) is distinct to that of FLA11 and FLA12 (type O) and constructs swapping either FAS11 or FAS3, into FLA12 were analysed. Plants with two transgene copies of *pFLA12*::FLA12-FAS11 and *pFLA12*::FLA12-FAS3 showed similar stem length as WT plants (**Figure 4A**; **Table 2**). Previous studies of FLA11 and FLA12 show location predominantly in the secondary wall and plasma membrane of fibre cells, respectively, as well as different degree of glycosylation in the AG2 region (Ma et al., 2022). The GPI-anchor and AG2 regions were swapped to generate plants containing *pFLA12*::FLA12-FLA11GPI, *pFLA12*::FLA12-FLA11linker+GPI, *pFLA12*::FLA12-FLA11AG2+linker+GPI and *pFLA12*::FLA12-FLA3AG+GPI constructs. Phenotyping of plants showed that *pFLA12*::FLA12-FLA11AG2+linker+GPI had shorter stem length compared to WT whereas all other constructs led to similar stem length as WT plants (**Figure 4A**; **Table 2**).

**Table 2:** Comparison of FAS1, AG2, and GPI domain swap plants^1^ stem length, lignin content and crystalline cellulose content at growth stage 6.9^2^.

Plant lines	Stem length (cm)	Lignin content (% AIR)	Crystalline cellulose content (% AIR)
WT	39.8 ± 3.4	12.0 ± 0.7	45.7 ± 3.9
OE-FLA12	39.0 ± 3.2	11.6 ± 2.4	46.2 ± 1.9
*pFLA12*::FLA12-FAS11	39.3 ± 3.5	11.1 ± 3.0	48.6 ± 1.8
*pFLA12*::FLA12-FAS3	40.5 ± 3.5	12.1 ± 2.1	48.6 ± 2.6
*pFLA12*::FLA12-FLA11GPI	40.8 ± 1.9	12.5 ± 1.2	40.9 ± 3.2*
*pFLA12*::FLA12-FLA11linker+GPI	40.9 ± 4.8	12.1 ± 2.2	45.9 ± 3.5
*pFLA12*::FLA12-FLA11AG2+linker+GPI	34.9 ± 4.1*	14.0 ± 2.8*	44.2 ± 4.9
*pFLA12*::FLA12-FLA3AG+GPI	42.7 ± 4.8	11.8 ± 1.8	45.7 ± 5.3

^1^ Transgenic plants with 1 transgene copy (1TC) were used. Data are shown as mean ± SD. N = 9 plants (for stem length) or 3 plants (for lignin and cellulose content) from three independent transformed lines. Asterisks indicates statistically significant difference, p < 0.05 using Student’s t-test.

^2^ Growth stages as outlined in Boyes et al. (2001).

See **Supplementary Figure S3** for schematic representation of domain swaps.

Mäule staining was further used to investigate cellular morphology in stem transverse sections and showed no obvious stem morphology or lignin composition changes of all transgene lines compared to WT (**Figures 4B–D**). Measurement of lignin content in mature stems of stage 6.9 (Boyes et al., 2001) showed that *pFLA12*::FLA12-FAS11, *pFLA12*::FLA12-FAS3, *pFLA12*::FLA12-FLA11GPI, *pFLA12*::FLA12-FLA11linker+GPI, and *pFLA12*::FLA12-FLA3AG+GPI plants similar to WT (**Table 2**). In contrast, *pFLA12*::FLA12-FLA11AG2+linker+GPI plants showed higher lignin content compared to WT (**Table 2**). Measurement of crystalline cellulose content in mature stems of stage 6.9 (Boyes et al., 2001) showed that only *pFLA12*::FLA12-FLA11GPI plants with a slight decrease compared to WT (**Table 2**). These results suggest that the AG2 domain of FLA12 is the major region that differentiates FLA12 functions from FLA11 as only OE-FLA12-FLA11AG2+linker+GPI plants showed reduced stem length and increased stem lignin content phenotypes compared to WT.

### 3.3 FLA12 location and trafficking are regulated by post-translational glycosylation

**Figure 5:**
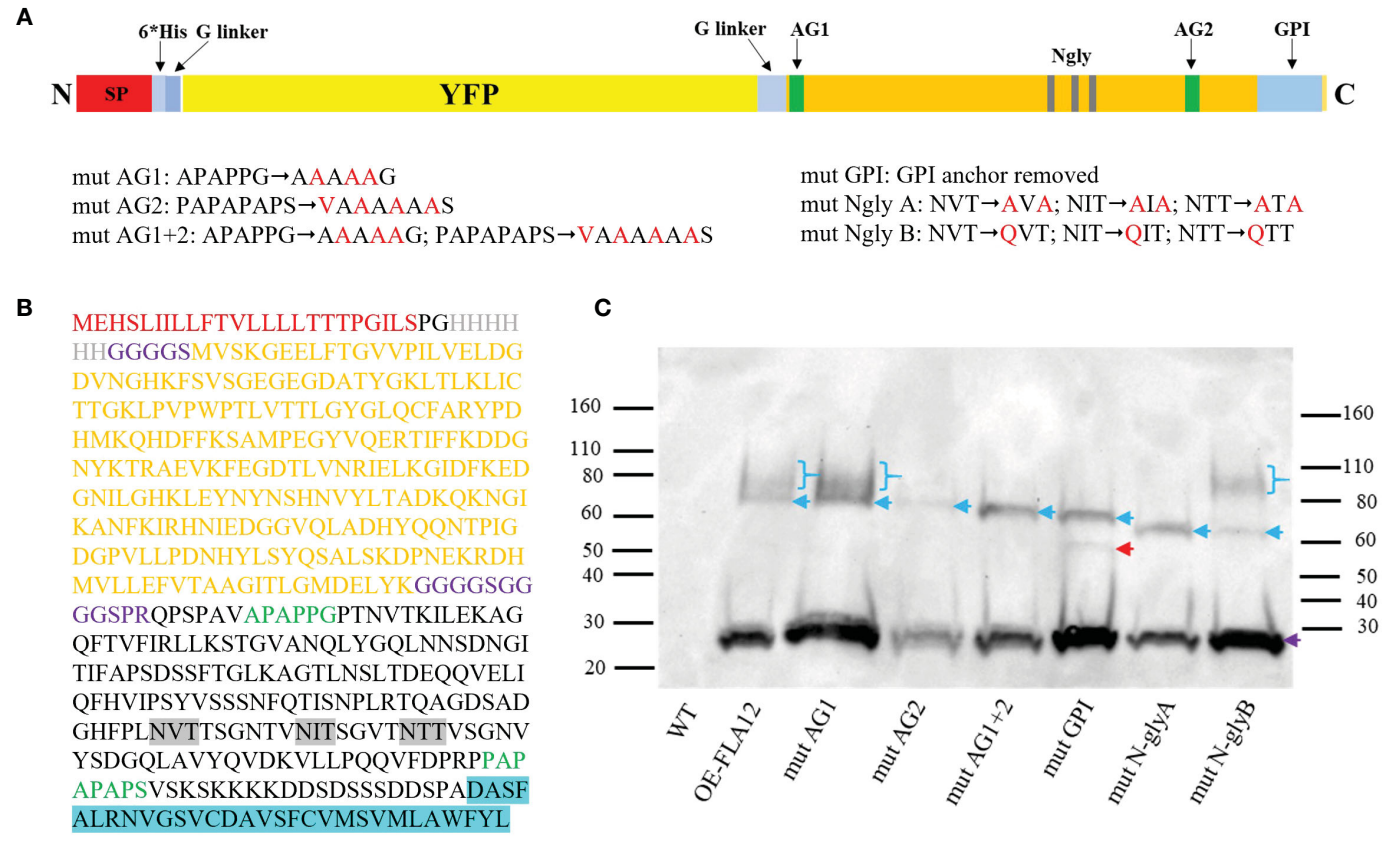
Schematic representation of FLA12 domain mutations and protein blotting analysis of YFP-FLA12 mutant variants. **(A)** Schematic YFP-FLA12 fusion protein and domain mutation designs. SP, signal peptide; 6*His, 6 Histidine repeat; G linker, Glycine linker; AG1, arabinogalactan motif 1; AG2, arabinogalactan motif 2; Ngly, *N*-glycosylation motif; GPI, glycosylphosphatidylinositol. **(B)** Amino acid sequences of YFP-FLA12 proteins. **(C)** Protein blotting analysis of OE-FLA12 and OE-FLA12 mutant variants. Denatured GFP-trap enriched proteins extracted from OE-FLA12 and mutant variants from 10-day-old seedlings were used for SDS-PAGE and protein blot analysis and detected with anti-YFP antibody. Blue arrows indicate YFP-FLA12 and mutant variant proteins. Bands with MW around 60 kDa are predicted YFP-FLA12 un-glycosylated proteins. Purple arrows indicated YFP cleaved from fusion proteins. Smeared bands of higher MW, indicated by blue brackets, likely represent proteins with AG glycosylation. Red arrow indicated a sharp band that was not identified in OE-FLA11mutGPI proteins as shown in previous studies (Ma et al., 2023). Molecular mass markers (kDa) were indicated on each side of the picture.

We have previously shown FLA12 plays a role regulating cellulose levels, likely in response to mechanical stress, whereas FLA11 regulates lignin levels and composition (Ma et al., 2022). The location of FLA12 is predominantly at the PM whereas FLA11 is largely released into the SCWs (Ma et al., 2022). The location of FLA12 at the cell surface and potential to act as a surface sensor led us to investigate FLA12 further. FLA12 mutant variants were generated to determine the influence on trafficking, location and biological functions. Mutant variants were generated in the AG glycomotifs that direct *O*-glycosylation at either the N-terminus (mutAG1), the C-terminal AG region (mutAG2) or both regions (mutAG1 + 2), variants with the GPI-anchor signal sequence removed (mutGPI), mutation of three predicted *N*-glycosylation motifs within the FAS1 domain by replacing NXS/T with AXA (mutNglyA, a non-conservative mutation which will prevent *N*-glycosylation or binding site function) and replacing NXS/T with QXS/T (mutNglyB, a conservative mutation which should retain function if a binding site) (**Figures 5A, B**).

In order to investigate the influence of these changes on post-translational modification, trafficking and location of FLA12, *pro35S*::YFP-FLA12 mutant variants were transiently expressed in *N. benthamiana* leaves. A previous study showed that tagRFP-FLA12 proteins located both at PM and cell wall of *N. benthamiana* leaf epidermal cells (Pinski et al., 2021). In this study, *N. benthamiana* leaf epidermal cells co-expressing each YFP-FLA12 mutant variant together with a Golgi marker (α-ManI-ECFP) and a PM marker (PIP2A-mCherry) were examined by fluorescence microscopy for the location of the fluorescence signals. YFP signals were observed at the cell wall, PM, and Golgi in plasmolyzed epidermal cells of leaves expressing YFP-FLA12 (**Supplementary Figure S4A**). YFP signal in cells expressing YFP-FLA12 were shown to largely overlay with the Golgi marker and a minor component with the PM marker. YFP signals in mutAG1, mutAG2 and mutAG1 + 2 fusion proteins showed similar location patterns to FLA12 (**Supplementary Figures S4B–D**). In contrast, YFP signals in mutGPI were shown to principally overlap with the PM marker (**Supplementary Figure S4E**). YFP signals in mutNglyA and mutNglyB were similar to FLA12 (**Supplementary Figures S4F–G**). The sub-cellular location of the FLA12 mutant variants supports the role of the GPI-anchor for correct trafficking and location of FLA12.

Transient expression of *pro35S*::YFP-FLA12 in *N. benthamiana* epidermal cells, which only contain primary walls, may not reflect the location of FLA12 in cells with secondary walls. Constructs containing the FLA12 mutated variants driven by the endogenous *FLA12* promoter were therefore stably transformed into WT Arabidopsis plants to enable investigation of FLA12 glycosylation and sub-cellular locations in stems. All transgenic plants carrying FLA12 mutations were confirmed with DNA sequencing and showed expression of YFP signals in XVs of seedling primary roots as expected for the *FLA12* promoter (**Supplementary Figure S5**). No obvious plant growth or stem length phenotypes were found between the OE-FLA12 mutant variants and either WT or OE-FLA12 plants of stage 8.0 and later (**Supplementary Figure S6**). Western blot analysis showed that all fusion proteins were expressed in comparable amounts (**Figure 5C**). OE-FLA12mutAG2, OE-FLA12mutAG1 + 2 and OE-FLA12mutN-glyA only showed sharp bands around 60 kDa, the expected size of HIS-YFP-FLA12 fusion proteins without glycosylation (**Figure 5C**). OE-FLA12, OE-FLA12mutAG1 and OE-FLA12mutN-glyB showed a smeared band around 80-110 kDa that likely corresponds to AG glycosylated proteins, suggests the main *O*-glycosylation occurs on AG2 (**Figure 5C**). OE-FLA12mutGPI showed two sharp bands around 50-60 kDa. The sharp bands in OE-FLA12mutN-glyA and OE-FLA12mutN-glyB had an obvious size shift (~5 kDa) to a smaller size, indicating that FLA12 is likely *N*-glycosylated (**Figure 5C**).

**Figure 6:**
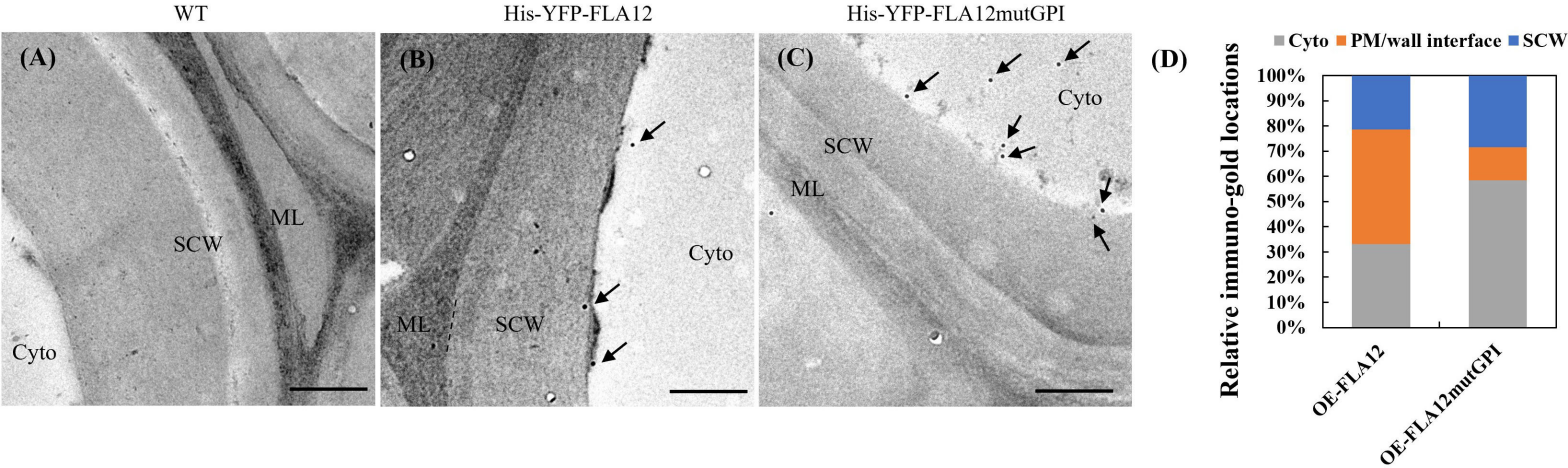
Sub-cellular location of FLA12 and FLA12mutGPI in stems at the base region of interfascicular fibre **(IF)** cells. TEM immunolabelling detection of HIS tagged FLA12 in ultrathin, transverse sections at 1 cm from the stem base at stage 6.9 (Boyes et al., 2001) IF cells from wild type (WT) control **(A)**, OE-FLA12 **(B)** and OE-FLA12mutGPI **(C)**. Arrow indicates gold particles. **(D)** Quantification of immuno-gold signals. FLA12 signals were found mostly retained at the interface between PM and secondary cell wall (SCW) in OE-FLA12 whereas OE-FLA12mutGPI was largely in the cytoplasm (cyto) of stem IF cells. ML: middle lamella. Scales = 500 nm.

Transient expression of *pro35S*::YFP-FLA12mutGPI in *N. benthamiana* indicated the GPI-anchor influences FLA12 trafficking and location (**Supplementary Figure S4**). The PM/wall was difficult to distinguish in the epidermal cells of *N. benthamiana* even with plasmolysis, therefore the effect of the GPI-anchor on FLA12 location in SCWs was investigated in stably transformed Arabidopsis plants in IF cells in stems using transmission-electron microscopy (TEM). Immuno-gold labelling was used to detect the HIS-tagged FLA12 in ultrathin transverse sections of stems in the base region at growth stage 6.9 (Boyes et al., 2001). In OE-FLA12 lines, immuno-gold labelling was detected mostly at the PM-wall (SCW) interface (**Figure 6**). In contrast, the immuno-gold labelling in mutGPI plants was present mostly in cytoplasm (**Figure 6**).

## 4 Discussion

SCWs of vascular tissues provide mechanical support for plant upright growth and transport of nutrients whilst simultaneously limiting tissue elongation. The initiation and development of SCWs is therefore carefully regulated. Cell surface sensing mechanisms are important for plant cells to coordinate development in response to environmental stimuli (Novaković et al., 2018). FLA11 and FLA12 were suggested to be cell surface sensors that regulate SCW development under mechanical stress (Ma et al., 2022). How FLA11 and FLA12 co-regulate SCW development remains unclear. In this study, we compared the promoter and domain features of FLA12 with FLA11, and the results suggest that the function of FLA12 is distinct from that of FLA11 during regulation of SCW development in leaves and stems.

*FLA*s belong to large gene families, with most diploid eudicots and monocots having around 20 members (Johnson et al., 2003; Johnson et al., 2017; Shafee et al., 2020). Both *FLA11*, *FLA12* and *FLA16* were suggested to be involved in SCW development (MacMillan et al., 2010; Liu et al., 2020; Ma et al., 2022). In this study, OE-FLA12 and OE-FLA12 mutant plants showed no obvious phenotypes, and *fla11*, *fla12* and *fla16* mutants show subtle phenotypes, suggesting functional redundancy. *FLAs* have been found to be highly expressed during tension wood development under mechanical tensile stress (Wang et al., 2017). Whether FLA12 functions predominantly under stress needs further investigation. The tissue specific expression patterns of FLAs have been well recognized, for example *FLA3* in reproduction tissue (Li et al., 2010), *FLA11* and *FLA12* in stems (MacMillan et al., 2010). The promoter regions of *FLA11* and *FLA12* showed that only *FLA12* can be potentially targeted by DNA-binding with one finger (DOF) transcription factors (Ma et al., 2022). Our domain swap experiments in this study suggested *FLA12* is predominately expressed in rosette leaves compared to *FLA11*. Two DOF TFs VDOP1 and VDOF2 were recently identified as negative factors in regulating leaf vein development and lignin biosynthesis (Ramachandran et al., 2020). It remains to be determined if VDOP1 and VDOF2 regulation of leaf vein development is via targeting *FLA12* and regulating the proportion of FLA12 and FLA11 in SCWs in leaves.

Domain swap experiments in this study (**Figure 4**), together with our previous findings (Ma et al., 2023), support the AG2 domain as the major region that differentiates functions between FLA11 and FLA12. Protein blots showed that the molecular mass of AG2 glycans of FLA11 (Ma et al., 2023) is slightly higher than FLA12 (**Figure 5**). Both the number of AG glycans added within AG motifs and/or the degree of polymerization of each AG glycan contribute to the molecular mass of the AG2 glycan. The contribution of AG glycans to the function of different FLAs remains to be determined. Recent studies showed that the terminal GlcA sugars of AGP may regulate calcium transporting and plant development (Lopez-Hernandez et al., 2020), suggests that terminal sugars between FLA11 and FLA12 glycans may also be important for their different functions. Deciphering the AG glycan structures of FLA12 and FLA11 will help uncover these fascinating mechanisms. Synthesis of AG glycans is a complex process that requires multiple glycosyl transferases (Ma et al., 2018). In this study, we showed that GPI-anchor signal sequence and *N*-glycosylation are both important for the synthesis of FLA12 AG2 glycans (**Figure 5**), which is also true for FLA11 as shown in our previous study (Ma et al., 2023). Thus, FLA11 and FLA12 also provide a system to investigate how different AG glycans are being synthesized and how other domains affect this process.

Bioinformatics analysis predicted that GPI-anchors are predominantly added to FLAs that belong to groups A and C (Shafee et al., 2020). Addition and modification of GPI-anchors in the ER/Golgi are proposed to promote (glyco)protein association with specific lipid microdomains, direct trafficking to the PM and facilitate interaction with signalling partners (Tapken and Murphy, 2015; Zavaliev et al., 2016; Yeats et al., 2018). Addition of GPI-anchors has been shown to direct proteins to specific regions of the cell, such as plasmodesmata and PM microdomains. GPI-anchors from two plasmodesmata located proteins, Callose Binding 1 (PDCB1) and β-1,3-glucanase (PdBG2), fused to two non-plasmodesmata localized proteins, AGP4 and LIPID TRANSFER PROTEIN1A was sufficient for targeting to plasmodesmata (Zavaliev et al., 2016). In this study, the GPI-anchor of FLA12 was shown to be required for location at the PM/cell wall interface (**Figure 6**). This suggests that FLA12 signalling partners are possibly co-located in close proximity to the PM, likely PM microdomains. Study of the GPI-anchored proteins LORELEI and LORELEI-like during reproduction development revealed they are essential for PM localization of the receptor kinase FERONIA that has been implicated in cell wall sensing in multiple pathways (Li et al., 2015; Liu et al., 2016). FLA12 has been implicated in regulating response to mechanical stress during secondary wall development and future analysis of the specific structures and roles of the GPI-anchor of FLA12 and identification of putative signalling components will bring new insights into FLA12 mechanisms during SCW development.

In summary, this study identified that FLA12 is predominantly expressed in leaf compared to FLA11, GPI-anchor is important for the cell surface location of FLA12. The AG2 glycans of FLA12 have different molecular mass compared to those of FLA11 and seem to be the major features that differentiate FLA12 and FLA11 functions. GPI-anchor signal sequence and *N*-glycosylation are also required for AG2 glycan synthesis. FLA12 may cooperate with FLA11 to regulate the time and degree of SCW development in different tissues and during different developmental stages of plant growth.

## Data availability statement

The original contributions presented in the study are included in the article/**Supplementary Material**. Further inquiries can be directed to the corresponding author.

## Author contributions

KJ: Conceptualization, Formal Analysis, Funding acquisition, Methodology, Project administration, Resources, Supervision, Writing – original draft, Writing – review & editing. YM: Conceptualization, Data curation, Formal Analysis, Investigation, Methodology, Validation, Writing – original draft, Writing – review & editing. JR: Data curation, Methodology, Visualization, Writing – review & editing. AB: Conceptualization, Funding acquisition, Supervision, Writing – review & editing.
