# Supplementary material for: Promoter and domain structures regulate FLA12 function during Arabidopsis secondary wall development
Source: Front Plant Sci. 2023 Nov 16;14:1275983. doi: 10.3389/fpls.2023.1275983 (PMC10687482; doi:10.3389/fpls.2023.1275983)
Supplement: Supplementary file 1 [file DataSheet_1.pdf]

Supplementary Material for:

Promoter and domain structures regulate FLA12 function during Arabidopsis secondary wall development

Yingxuan Ma, Julian Ratcliffe, Antony Bacic, Kim L. Johnson*

*** Correspondence:**
Kim L. Johnson
k.johnson@latrobe.edu.au

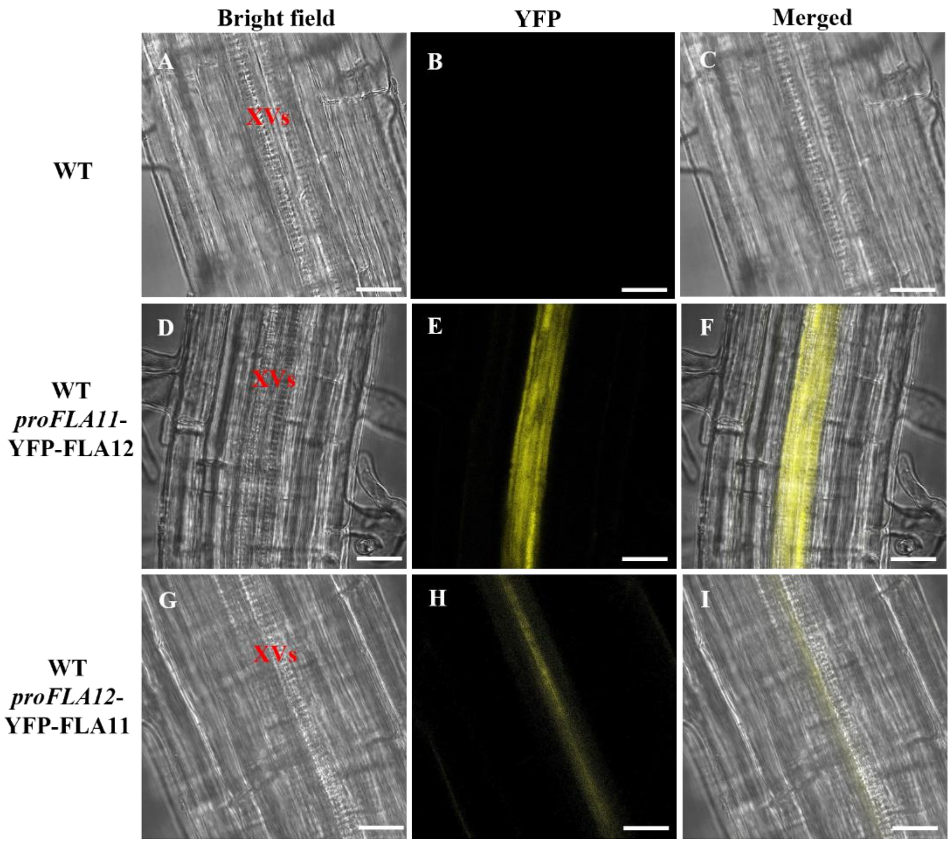

Supplementary Fig. S1. Visualization of YFP fluorescence signals in roots of *proFLA11*::YFP-FLA12 and *proFLA12*::YFP-FLA11 10-day old seedlings. YFP fluorescence signals were observed in seedling root xylem vessels (XVs). Scales = 20 µm.

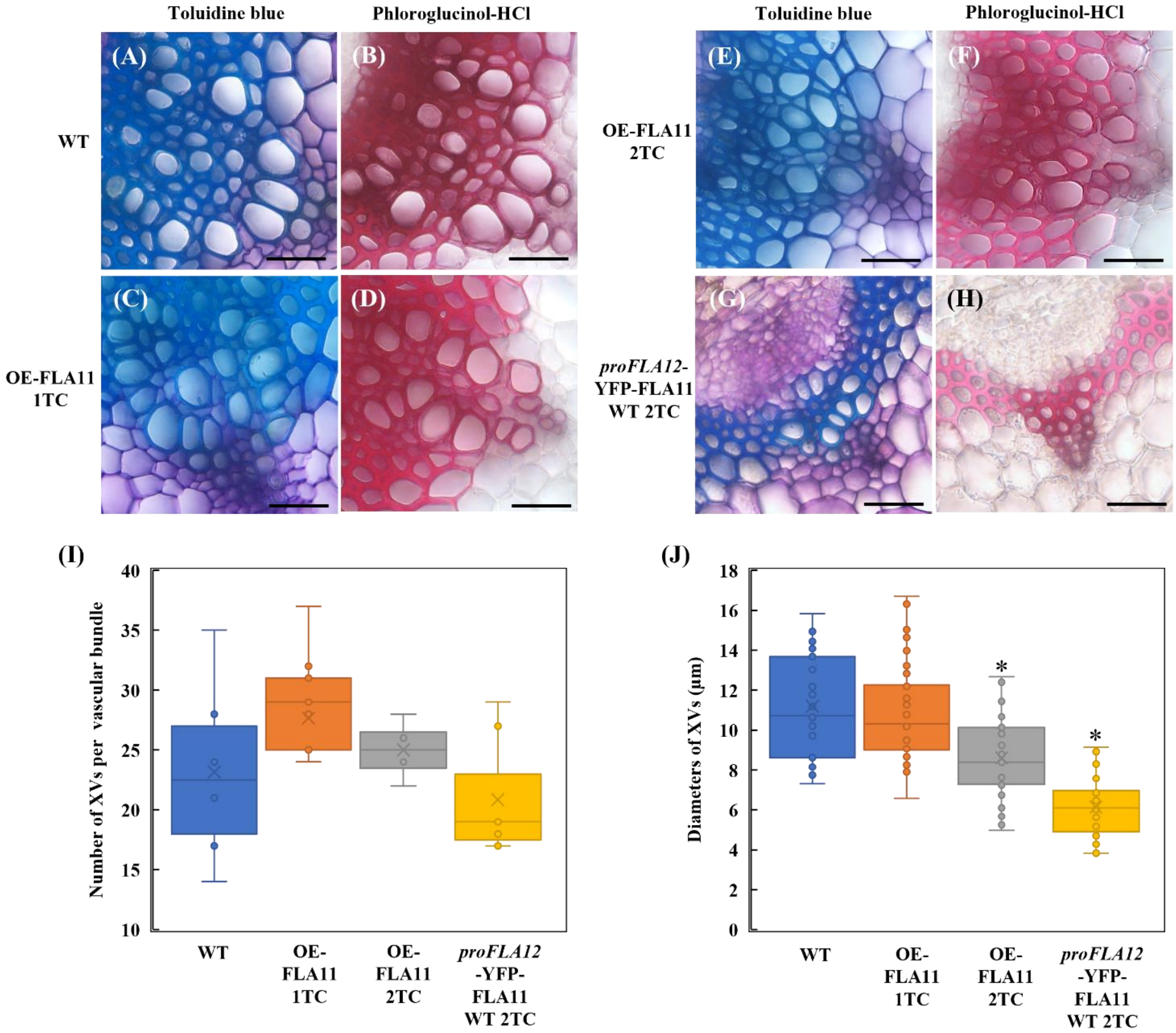

Supplementary Fig. S2. Histology of mature stems of WT, OE-FLA11, and *proFLA12*::YFP-FLA11 WT plants. Fresh stems of WT **(A-B)**, OE-FLA11 with one transgene copy (1TC) **(C-D)**, OE-FLA11 with two transgene copies (2TC) **(E-F)**, and *proFLA12*::YFP-FLA11 WT 2TC **(G-H)** plants at growth stage 6.9 (Boyes et al., 2001) were sectioned at 1 cm above the stem base and stained with Toluidine blue O or phloroglucinol-HCl. **(I)** Quantification of the number of xylem vessels (XVs) per vascular bundle in stems showed no significant differences between all lines. **(J)** The diameter of XVs in *proFLA12*::YFP-FLA11 WT 2TC was significantly smaller compared to WT and OE-FLA11. Scale bar = 20 µm.

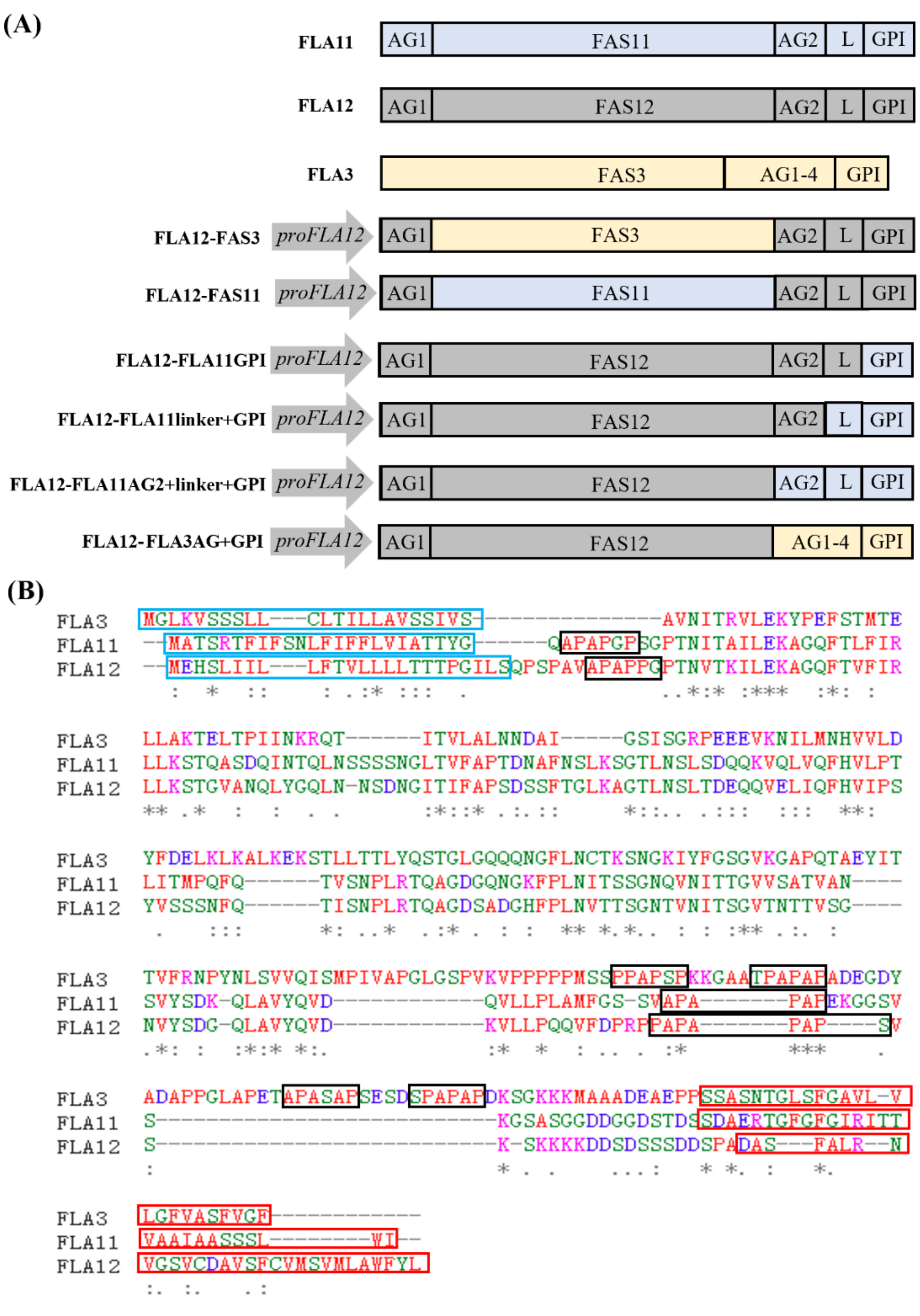

Supplementary Fig. S3. Schematic representation of FLA12 domain swaps and alignment of FLA3, FLA11, and FLA12 protein sequences. (A) FLA11, FLA12 and FLA3 proteins shaded in blue, grey, and yellow, respectively. AG1: AG glycomotif at N-terminal. AG2: AG glycomotif closest to GPI-anchor region (GPI) at C-terminal. AG1-4: AG glycomotif 1 to 4 of FLA3. FAS11: FAS1 region of FLA11. FAS12: FAS1 region of FLA12. FAS3: FAS1 region of FLA3. **(B)** Alignment of FLA3, FLA11 and FLA12 protein sequences. Protein alignment was performed using online tools CLUSTAL O(1.2.4) multiple sequence alignment at EMBL-EBI with default parameters. AG domains were indicated by black rectangles, signal peptides were indicated by blue rectangles, GPI-anchor signal sequences were indicated by red rectangles.

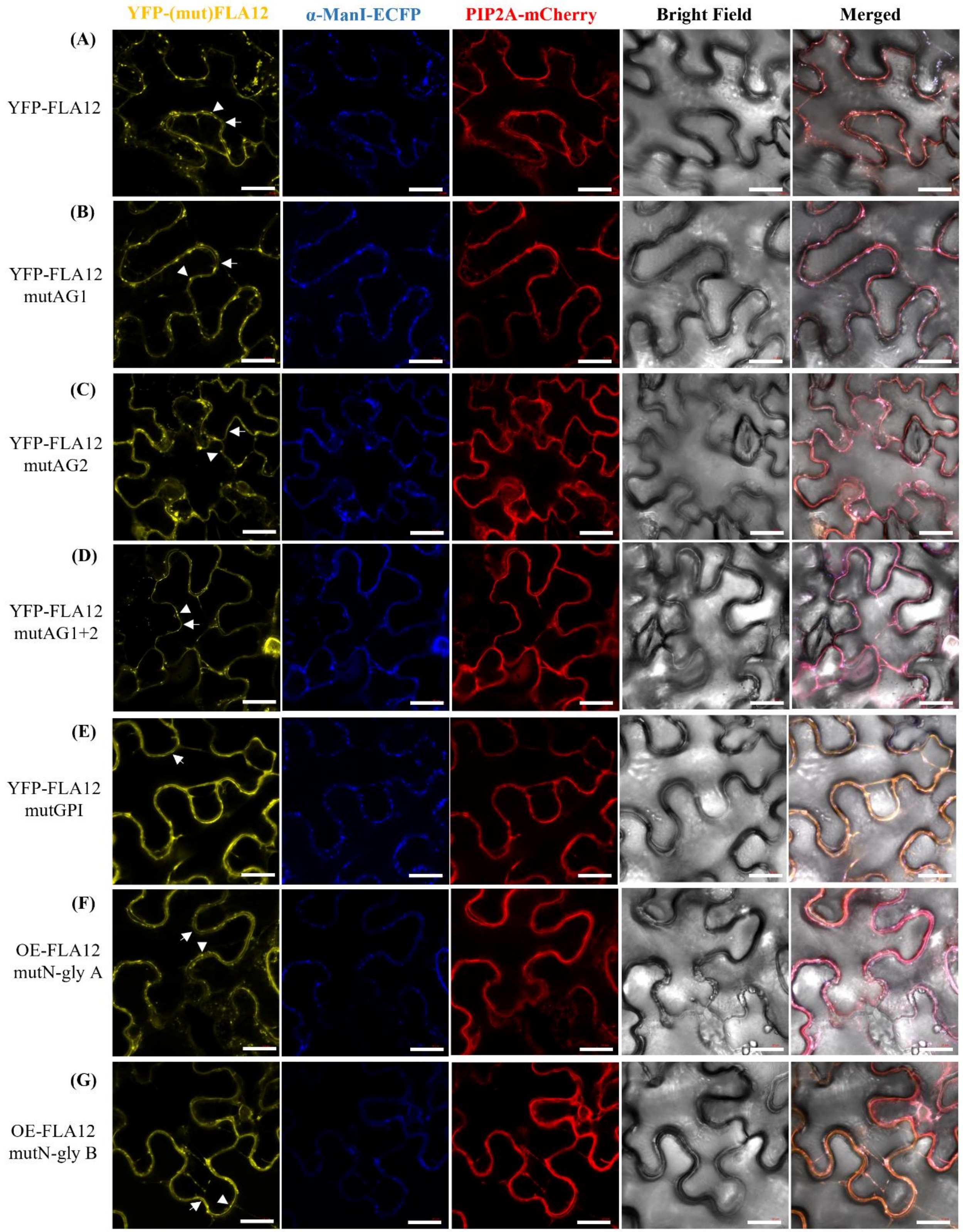

Supplementary Fig. S4. Transient expression and protein localization of YFP-FLA12 and YFP-FLA12 mutant variants in *N. benthamiana* leaves. (A) YFP-FLA12 co-expressed with Golgi

marker (α-ManI-ECFP) and plasma membrane (PM) marker (PIP2A-mCherry) and observed with confocal microscopy. Location of YFP-FLA12 mut N-terminal AG1 motif **(B),** YFP-FLA12 mut C-terminal AG2 motif **(C)**, YFP-FLA12 mut AG1+2 motifs (**D**), YFP-FLA12 mut GPI motif **(E)**, YFP-FLA12 mut NglyA **(F),** YFP-FLA12 mut NglyB **(G)**. Arrowheads in YFP channel indicate co-location of YFP-FLA12 with PIP2A-mCherry, arrows indicate co-location of YFP-FLA11 with α-Manl-ECFP. Scales = 20 µm.

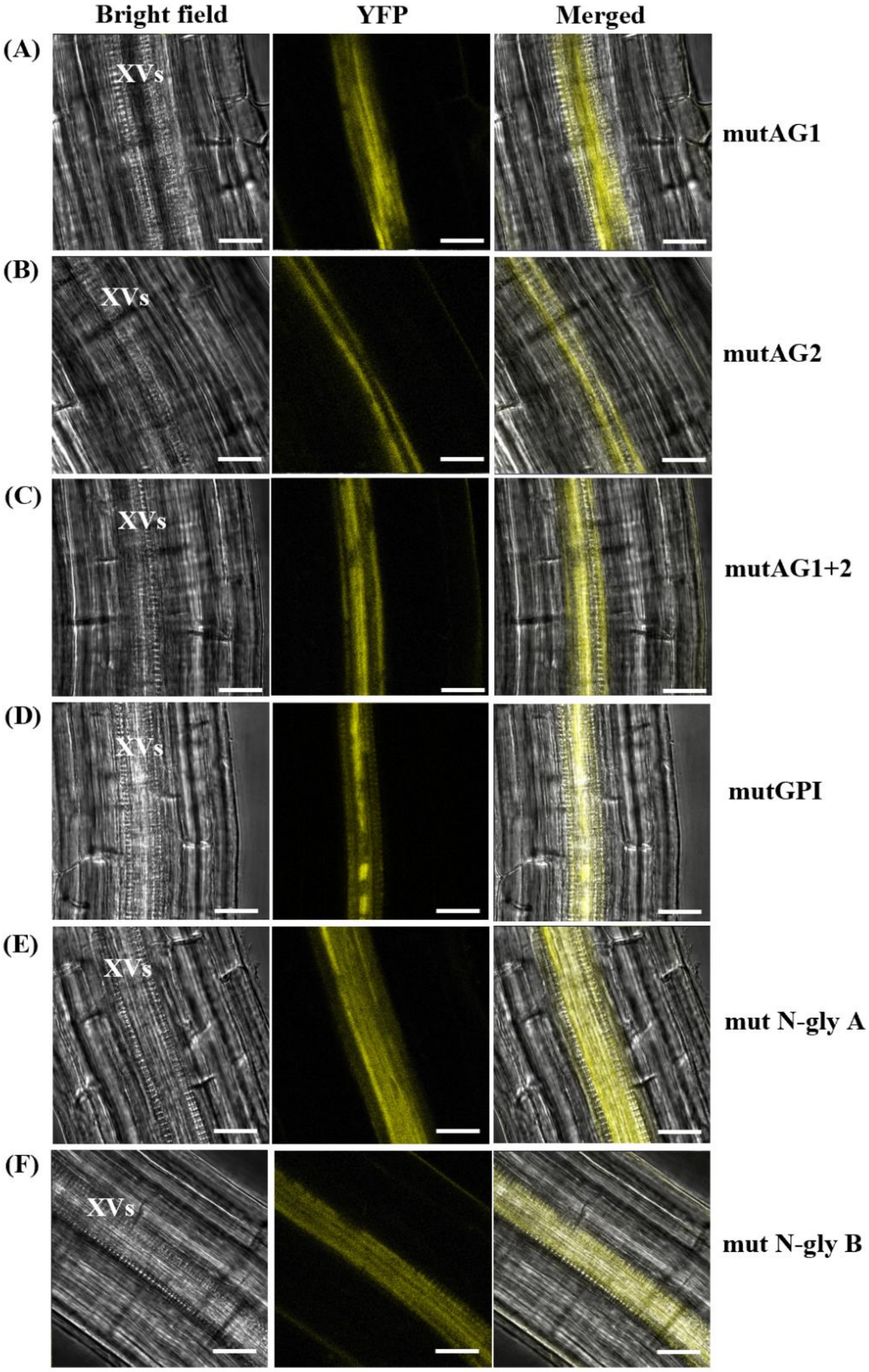

Supplementary Fig. S5. Visualization of fluorescence signals of YFP-FLA12 mutant variant proteins in 10-day old seedling roots. YFP fluorescence signals observed in xylem vessels (XVs) in roots of OE-FLA12mutAG1 **(A)**, OE-FLA12mutAG2 **(B)**, OE-FLA12mutAG1+2 **(C)**, OE-FLA12mutGPI **(D)**, OE-FLA12mutNglyA **(E)** and OE-FLA12mutNglyB **(F)**. Scales = 20 µm.

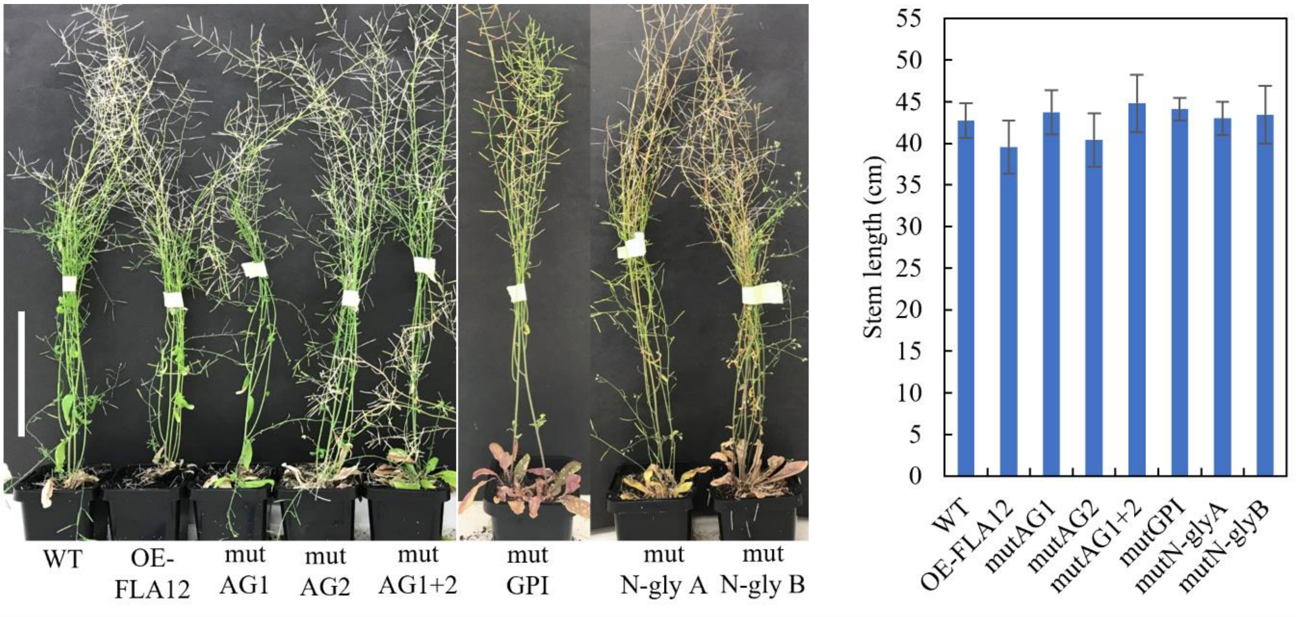

Supplementary Fig. S6. Growth phenotypes of wild type (WT) and OE-FLA12 mutant variant plants. Phenotypes of mature plants at growth stage 8.0 and later (Boyes et al., 2001) show similar plant morphology in WT, OE-FLA12 and OE-FLA12 mutant variant transgenic plants. Measurement of stem length show no obvious difference between WT, OE-FLA12 and OE-FLA12 mutant variant transgenic plants. Data shown as average ± SD. N ≥ 6 plants from three independent transformed lines. Scale = 10 cm.

Supplementary Table S1. List of FLA12 domain mutation and deletion vectors used.

Vector ID	Description	Purpose
YMV201	pGreen0179-*35S*-spFLA12-His-YFP-FLA12	Tobacco transient expression.
YMV202	pGreen0179-*35S* -spFLA12-His- YFP -FLA12 AG1mutation	
YMV203	pGreen0179-*35S* -spFLA12-His- YFP -FLA12 AG2 mutation	
YMV204	pGreen0179-*35S* -spFLA12-His- YFP -FLA12 AG1+2 mutation	
YMV205	pGreen0179-*35S* -spFLA12-His- YFP -FLA12 no GPI	
YMV208	pGreen0179-*35S* -spFLA12-His-YFP-FLA12 NglyA mutation	
YMV209	pGreen0179-*35S* -spFLA12-His-YFP-FLA12 NglyB mutation	
YMV211	pGreen0179-*proFLA12*-spFLA12-His-YFP-FLA12	Transgenic Arabidopsis plants.
YMV212	pGreen0179-*proFLA12*-spFLA12-His- YFP -FLA12 AG1 mutation	
YMV213	pGreen0179-*proFLA12*-spFLA12-His- YFP -FLA12 AG2 mutation	
YMV214	pGreen0179-*proFLA12*-spFLA12-His- YFP -FLA12 AG1+2 mutation	
YMV215	pGreen0179-*proFLA12*-spFLA12-His- YFP -FLA12 no GPI	
YMV218	pGreen0179-*proFLA12*-spFLA12-His-YFP-FLA12 NglyA mutation	
YMV219	pGreen0179-*proFLA12*-spFLA12-His-YFP-FLA12 NglyB mutation	

Supplementary Table S2. List of primers used for FLA12 domain mutation and deletion vectors construction.

Name	Primer sequence	Purpose
YMV211-proF12-F	ACTATAGGGCGAATTGGGTACCctcggaaaatagggctttttg	Cloning *FLA12* promoter
YMV211-proF12-R	GATGATGAGAGAATGTTCCATgggctctgttttcaagagtgttgatat	
pGreenKpnI35S-F	CGACTCACTATAGGGCGAATTG	FLA12 AG1 mutation vector construction
F12-AG Mut1_1R	TCCTGCTGCGGCTGCAGCGACGGCTGGAGAGGGCTG	
F12-AG Mut1_2F	GCTGCAGCCGCAGCAGGACCCACAAACGTTACCAAA	
pGreenNotIOCS-R	CTGGAGCTCCACCGCG	
pGreenKpnI35S-F	CGACTCACTATAGGGCGAATTG	FLA12 AG2 mutation vector construction
F12-AG Mut2_1R	CGCAGCCGCAGCCGCAGCAACAGGACGAGGATCGAAAACTTGTTGTG	
F12-AG Mut2_2F	GTTGCTGCGGCTGCGGCTGCGTCTGTATCGAAATCAAAGAAGAAGAAG	
pGreenNotIOCS-R	CTGGAGCTCCACCGCG	
pGreenKpnI35S-F	CGACTCACTATAGGGCGAATTG	FLA12 AG1+2 mutation vector construction
F12-AG Mut1_1R	TCCTGCTGCGGCTGCAGCGACGGCTGGAGAGGGCTG	
F12-AG Mut1_2F	GCTGCAGCCGCAGCAGGACCCACAAACGTTACCAAA	
F12-AG Mut2_1R	CGCAGCCGCAGCCGCAGCAACAGGACGAGGATCGAAAACTTGTTGTG	
F12-AG Mut2_2F	GTTGCTGCGGCTGCGGCTGCGTCTGTATCGAAATCAAAGAAGAAGAAG	
pGreenNotIOCS-R	CTGGAGCTCCACCGCG	
pGreenKpnI35S-F	CGACTCACTATAGGGCGAATTG	FLA12 GPI deletion vector construction
YM205F11noGPI_R	CATATCTCATTAAAGCAGGACTCTAGATCACGCCGGAGAATCATCACTG	
YM205F11noGPI_F	TCTAGAGTCCTGCTTTAATGAGATATG	
pGreenNotIOCS-R	CTGGAGCTCCACCGCG	
pGreenKpnI35S-F	CGACTCACTATAGGGCGAATTG	FLA12 N-GlyA mutation vector construction
F12-NglcA-Mu_1R	tgcagaagcGAGCTGAGTGTTGATTTGGTCTG	
F12-NglcA-Mu_2Fnew	ATCAACACTCAGCTCgcttctgcaTCGAGTAATGGCTTAACCGTGT	
F12-NglcA-Mu_2R	tgcgatcgcAACTTGGTTACCGGAGCTagcgatggcAAGAGGGAATTTACCGTTTTGG	
F12-NglcA-Mu_3F	gccatcgctAGCTCCGGTAACCAAGTTgcgatcgcaACTGGAGTTGTCAGCGCCAC	
pGreenNotIOCS-R	CTGGAGCTCCACCGCG	
F12-NglcA-Mu_1R	tgcagaagcGAGCTGAGTGTTGATTTGGTCTG	FLA12 N-GlyB mutation vector construction
F12-NglcB-Mu_1R	ttgGAGCTGAGTGTTGATTTGGTCTG	
F12-NglcB-Mu_2Fnew	ACCAAATCAACACTCAGCTCcaaTCTTCCTCGAGTAATGGCTTAAC	
F12-NglcB-Mu_2R	ttgAACTTGGTTACCGGAGCTAGTGATctgAAGAGGGAATTTACCGTTTTGG	
F12-NglcB-Mu_3F	cagATCACTAGCTCCGGTAACCAAGTTcaaATCACCACTGGAGTTGTCAGC	
pGreenNotIOCS-R	CTGGAGCTCCACCGCG	

Supplementary Table S3. List of vectors used for domain swaps.

Vector ID	Description	Purpose
YMV161	pGreen0179-*proFLA12*::spFLA11-His-YFP-FLA11	Promoter swap study
YMV273	pGreen0179-*proFLA12*::spFLA12-His-YFP-FLA12AG1-FAS11-FLA12AG2-FLA12GPI	FAS1 swap study
YMV272	pGreen0179-*proFLA12*::spFLA12-His-YFP-FLA12AG1-FAS3-FLA12AG2-FLA12GPI	
YMV262	pGreen0179-*proFLA12*::spFLA12-His-YFP-FLA12AG1-FAS12-FLA12AG2-FLA12 linker-FLA11GPI	AG2 and GPI swap study
YMV276	pGreen0179-*proFLA12*::spFLA12-His-YFP-FLA12AG1-FAS12-FLA12AG2-FLA11 linker-FLA11GPI	
YMV275	pGreen0179-*proFLA12*::spFLA12-His-YFP-FLA12AG1-FAS12-FLA11AG2-FLA11 linker-FLA11GPI	
YMV274	pGreen0179-*proFLA12*::spFLA12-His-YFP-FLA12AG1-FAS12-FLA3AG-FLA3GPI	

Supplementary Table S4. List of primers used for domain swap vector constructs.

Name	Primer sequence	Purpose
YMV273-FAS11-F	CAGGCGGAGGTGGGTCAcctaggCAGCCCTCTCCAGCCGTCGCTCCGGCCCCACCAGGACCAACGAACATAACCGCAATCCTAG	YMV273
YMV273-FAS11-R	CAAAACTTGATCAACCTGATAAACGGCC	
YMV273-F12AG-F	ATCAGGTTGATCAAGTTTTGCTTCCACAACAAGTTTTCGATCCTCG	
pGreen-OCS-R	CTGGAGCTCCACCGCG	
YMV272-FAS3-F	CAGGCGGAGGTGGGTCAcctaggCAGCCCTCTCCAGCCGTCGCTCCGGCCCCACCAGGAGTTAACATAACCCGAGTGCTCG	YMV272
ARP4-FAS3-R	TTTCACACCAGAACCAAAGTAAATC	
YMV272-F12AG_F	ACTTTGGTTCTGGTGTGAAACTTCCACAACAAGTTTTCGATCCTCG	
pGreen-OCS-R	CTGGAGCTCCACCGCG	
YMV211proF12-F	ACTATAGGGCGAATTGGGTACCctcggaaaatagggctttttg	YMV262
262F12-R	AACGGTAGTGATTCTGATCCCAAACCCGAATCCAGTCCTCTCTGCATCAGACGCCGGAGAATCATCACTG	
262F11GPI-F	GGATCAGAATCACTACCGTTGCAGCCATTGCTGCTTCTTCTTCTCTGTGGATATAATCTAGAGTCCTGCTTTAATGAGAT	
pGreen-OCS-R	CTGGAGCTCCACCGCG	
YMV201-F12-F	CAGGCGGAGGTGGGTCAcctaggCAGCCCTCTCCAGCCGTC	YMV276
YMV276-FAS12-AG2-R	AGACGGAGCCGGAGCC	
YMV276-F11link-GPI-F	CTCCGGCTCCGGCTCCGTCTGAGAAAGGCGGCTCTGTTTCAAAAGG	
pGreen-OCS-R	CTGGAGCTCCACCGCG	
YMV201-F12_F	CAGGCGGAGGTGGGTCAcctaggCAGCCCTCTCCAGCCGTC	YMV275
YMV171-FAS12_R	CAAAACCTTATCGACCTGATAAACAGCG	
YMV171-F11AG_F	ATCAGGTCGATAAGGTTTTGCTGCCATTAGCCATGTTTGGATCAAG	
pGreen-OCS-R	CTGGAGCTCCACCGCG	
YMV274-FAS12_F	GTTCAGGCGGAGGTGGGTCAcctaggCCCACAAACGTTACCAAAATCCTAGAG	YMV274
YMV171-FAS12_R	CAAAACCTTATCGACCTGATAAACAGCG	
YMV274-F3AG-GPI_F	ATCAGGTCGATAAGGTTTTGGGCGCTCCTCAAACCGC	
pGreen-OCS-R	CTGGAGCTCCACCGCG	
